# C-terminal domain small phosphatase-like 2 promotes epithelial-to-mesenchymal transition via Snail dephosphorylation and stabilization

**DOI:** 10.1098/rsob.170274

**Published:** 2018-04-04

**Authors:** Yulan Zhao, Jinquan Liu, Fenfang Chen, Xin-Hua Feng

**Affiliations:** 1 Life Sciences Institute, and Innovation Center for Cell Signaling Network, Zhejiang University, Hangzhou, Zhejiang 310058, People's Republic of China; 2 Michael E. DeBakey Department of Surgery, Baylor College of Medicine, Houston, TX 77030, USA; 3 Department of Molecular and Cellular Biology, Baylor College of Medicine, Houston, TX 77030, USA

**Keywords:** Snail, CTDSPL2/SCP4, phosphatase, TGF-β signalling, phosphorylation, EMT

## Abstract

The epithelial-to-mesenchymal transition (EMT) is a cellular reprogramming process converting epithelial cells into mesenchymal cell morphology. Snail is a critical regulator of EMT by both suppressing epithelial gene expression and promoting mesenchymal gene expression. Expression and activity of Snail are tightly controlled at transcriptional and post-translational levels. It has previously been reported that Snail undergoes phosphorylation and ubiquitin-dependent proteasome degradation. Here, we report nuclear phosphatase SCP4/CTDSPL2 acts as a novel Snail phosphatase. SCP4 physically interacts with and directly dephosphorylates Snail. SCP4-mediated dephosphorylation of Snail suppresses the ubiquitin-dependent proteasome degradation of Snail and consequently enhances TGFβ-induced EMT. The knockdown of SCP4 in MCF10A mammary epithelial cells leads to attenuated cell migration. Collectively, our finding demonstrates that SCP4 plays a critical role in EMT through Snail dephosphorylation and stabilization.

## 1. Introduction

The epithelial–mesenchymal transition (EMT) is the process whereby epithelial cells lose cell polarity and cell–cell contacts, and undergo drastic morphological changes, acquiring highly migratory and invasive properties [1–5]. It is thought to be critical for many developmental events such as gastrulation and neural crest migration. A similar process is also activated during wound healing and the development of organ fibrosis [1,6]. During tumour progression, EMT is found to provide tumour cells with the ability to migrate and invade adjacent tissues, which accounts for approximately 90% of human cancer deaths [7,8]. One of the hallmarks of EMT is functional loss of E-cadherin, a metastatic suppressor during tumour progression [9]. Downregulation of E-cadherin is an essential step towards the invasive phase of carcinoma, and in many metastatic cancers, which is largely due to transcriptional repression [10].

Several transcriptional repressors, including Snail/Slug family, the bHLH family (E47 and twist), two ZEB factors (ZEB1 and SIP) and FOXC2, respond to different microenvironmental stimuli and function as molecular switches of EMT [11–14]. Their elevated expression has been well documented in invasive tumours. These factors repress the expression of E-cadherin, as well as other epithelial markers, but also enhance the expression of mesenchymal genes. Among these, the zinc-finger protein Snail plays an important role in both embryonic development and cancer metastasis [15,16]. Snail binds to the E-box region of its target gene promoters through a C-terminal zinc-finger domain and represses their transcription by associating with histone deacetylase activity through its N-terminal region [16,17]. It has been well established that enhancement in Snail expression in primary tumours promotes cellular motility and acquisition of metastatic properties [18]. In non-transformed cells, the enhancement in Snail protein expression induces fibrosis-like features [19], while targeted downregulation of Snail can reverse EMT [20].

While induction of Snail transcription precedes EMT induction, post-translational regulation of Snail is also critical for determining Snail's protein level or stability, and capacity to induce EMT [21]. Snail is a labile protein with a short half-life about 25 min, which is tightly regulated by the ubiquitin–proteasome pathway. To date, several studies have illustrated the post-translational modifications that control Snail function. For example, GSK3β binds to and phosphorylates Snail at two consensus motifs to dually regulate β-TrCP-mediated ubiquitination and subcellular localization [21]. In addition, CK1ε, another protein kinase, is required for the subsequent GSK3β phosphorylation of Snail [22]. Furthermore, Lats2 interacts with Snail and phosphorylates Snail at residue T203, and serves to retain Snail in the nucleus [23]. The small C-terminal domain phosphatase-1, 2 and 3 (collectively called SCP1/2/3), which are small phosphatase members of the FCP/SCP phosphatase family, were first identified to interact with and dephosphorylate Snail at the GSK3β phosphorylation motif and regulate its stability and localization [24]. However, additional Snail-specific phosphatase(s) remain to be identified.

CTDSPL2 (also named SCP4) is a distant member of the FCP/SCP phosphatases. SCP4 specifically dephosphorylates Smad1, 5 and 8, but not Smad2 and Smad3, and as a consequence attenuates the BMP signalling pathway, but not the TGFβ signalling pathway [25]. During the course of these studies, we found that SCP4 promoted EMT, apparently independent of TGFβ signalling. In this study, we reported that SCP4 acts as a novel nuclear phosphatase for Snail. Increased expression of SCP4 promoted TGFβ-induced EMT and, conversely, depletion of SCP4 attenuated TGFβ-induced migration and EMT. Furthermore, we demonstrated SCP4 physically interacted with and specifically dephosphorylated Snail at the GSK3β phosphorylation motif. SCP4 prolonged the half-life of Snail by inhibiting its phosphorylation, ubiquitination and degradation. Hence, SCP4-mediated dephosphorylation of Snail provides an additional mechanism in EMT and metastasis.

## 2. Material and methods

### 2.1. Mammalian expression plasmids

Expression plasmids for full-length SCP4, its phosphatase-dead mutant (D293E, D295N) and deletion mutants were obtained by PCR and cloned into EcoRI (5′) and SalI (3′) of pXF6F (a derivative of pRK5, Genentech). Their sequence integrity was confirmed by sequencing. Plasmids expressing wild-type Snail and its mutant Snail-S2A, Snail-S4A, Snail-S6A and Snail-2A4E were obtained from Dr Mien-Chie Hung.

### 2.2. Antibodies and reagents

Antibodies against Snail, Slug, E-cadherin, vimentin, HA and SCP4 (Cell Signaling Technology), β-actin, γ-tubulin and FLAG tag (Sigma), N-cadherin (BD Biosciences) and ubiquitin (Santa Cruz) were obtained commercially. MG132 was purchased from Merck. Puromycin was purchased from Sigma.

### 2.3. Cell culture, cell transfection, immunoprecipitation and western blotting

HEK293T and MCF10A cells were cultured and transfected using Lipofectamine (Invitrogen) as previously described [26]. Generation of MCF10A stable cells expressing HA-tagged SCP4 or SCP4DN mutant was performed as previously described [26].

Immunoprecipitation (IP) was carried out as previously described [26]. HEK293T cells were transiently transfected with expression plasmids for HA–Snail and FLAG–SCP4. Twenty-four hours after transfection, cell lysates were harvested by Myc lysis buffer (137 mM NaCl, 20 mM Tris–HCl (pH 8.0), 1% Non-idet P-40) and anti-FLAG antibodies were used to immunoprecipitate FLAG–SCP4 from transfected cell lysates by incubating with protein A Sepharose CL-4B (GE Healthcare) at 4°C for 4 h. After extensive washes, immunoprecipitated proteins were separated by SDS–PAGE, transferred onto polyvinylidene difluoride membrane and immunostained with anti-Myc or anti-FLAG antibodies, and finally detected by horseradish peroxidase-conjugated secondary antibodies and visualized by chemiluminescence (Pierce).

#### 2.3.1. *In vitro* GST pull-down assay

*In vitro* protein translation was performed from the pRK5-derived vector using SP6 RNA polymerase and the TNT Quick Coupled Transcription/Translation System (Promega). Proteins fused with GST in pGEX vector were expressed in *Escherichia coli* BL21 (DE3) strain and purified according to the manufacturer's instructions. GST pull-down experiments were carried out as previously described [27].

### 2.4. RNA interference

Small interference siRNAs targeting human SCP4, i.e. siSCP4, were made by RiboBio Co (#1 target sequence: nt 1361–1379 of coding region, GAGACAGATTTCGCTTGCA; #2 target sequence: nt 1009–1027 of coding region, GAACGAATGTCTCAGATGT; #3 target sequence: nt 619–637 of coding region, GTGAGACCATCACTAAACA). Cells were transfected with siControl or siSCP4 using Lipofectamine RNAiMAX (Invitrogen).

### 2.5. Lentivirus production and stable cell line generation

SCP4 or mutant SCP4DN cDNA was subcloned into pWPI-puro vector at EcoRI and PmeI sites to generate pWPI-SCP4 or pWPI-SCP4DN. HEK293T cells were transfected with pWPI-SCP4 or pWPI-SCP4DN together with lentiviral packaging plasmid psPAX2 and envelope plasmid pMD2.G. After 48 h culture, lentiviruses were collected from medium, purified by centrifuge and then used to infect host cells. Stable cells were selected in the presence of 2 ng ml^−1^ of puromycin.

### 2.6. Quantitative RT–PCR

Total RNAs were extracted using TRIzol (Invitrogen). One microgram of total RNAs was reverse transcribed to complementary DNA using PrimeScript RT reagent kit (TaKaRa). Quantitative RT–PCR (qRT–PCR) was performed using SyBR green (Applied Biosystems) with β-actin as an internal loading control on an ABI PRISM 7500 Sequence Detector System (Applied Biosystems). Samples were done in triplicate and data were analysed using the 2^−ΔCT^ method. Primers used for specific mouse genes are listed as below: E-cadherin, 5′-CGGGAATGCAGTTGAGGATC-3′ (forward) and 5′-AGGATGGTGTAAGCGATGGC-3′ (reverse); N-cadherin, 5′-ACCAGGTTTGGAATGGGACAG-3′ (forward) and 5′-ATGTTGGGTGAAGGGGTGCTTG-3′ (reverse); vimentin, 5′-TGAAGGAGGAAATGGCTCGTC-3′ (forward) and 5′-GTTTGGAAGAGGCAGAGAAATCC-3′ (reverse); fibronetin, 5′-TGAAAGACCAGCAGAGGCATAAG-3′ (forward) and 5′-CTCATCTCCAACGGCATAATGG-3′ (reverse); Snail, 5′-ATCGGAAGCCTAACTACAGCGAGC-3′ (forward) and 5′- CAGAGTCCCAGATGAGCATTGG-3′ (reverse); β-actin, 5′-TGAGCGCAAGTACTCTGTGTGGAT-3′ (forward) and 5′-ACTCATCGTACTCCTGCTTGCTGA-3′ (reverse).

### 2.7. Wound-healing assay

MCF10A cells were seeded in a six-well plate and allowed to grow to nearly 100% confluence in culture medium. Subsequently, a cell-free line was manually created by scratching the confluent cell monolayers with a 200 µl pipette tip. The wounded cell monolayers were washed twice with PBS and incubated in OPTI-MEM medium with 1 ng ml^−1^ of TGFβ alone or in combination with inhibitor SB431542 for the indicated time periods.

### 2.8. Transwell assay

The transwell assay was performed using Transwell inserts (BD Bioscience). 1 × 10^5^ cells were seeded into an insert with 8.0 µm pore size. We then added 500 µl of complete cell culture medium into the bottom well (under the insert) for incubation at 37°C and 5% CO_2_. After 8 h incubation, cells were fixed, stained with DAPI for 10 min and microscopically analysed.

### 2.9. Statistical analysis

Results were shown as means ± s.e.m. All experiments were repeated at least three times. The mean values were compared with controls by Student's *t*-test.

## 3. Results

### 3.1. Ectopic expression of SCP4 enhances TGFβ-induced epithelial–mesenchymal transition and promotes migration in MCF10A

**Figure 1.**
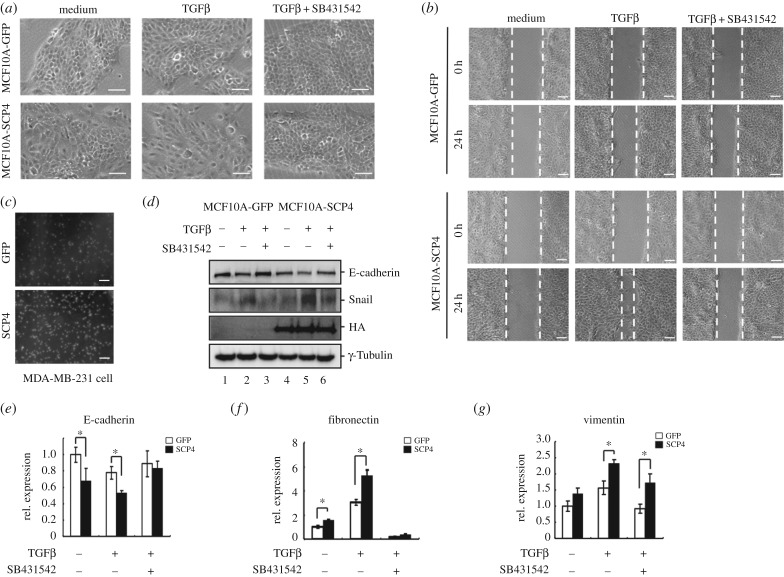
Ectopic expression of SCP4 enhances TGFβ-induced EMT and promotes migration in MCF10A. (*a*) SCP4 promotes TGFβ-induced morphological changes. MCF10A cells stably expressing SCP4 or GFP were treated with TGFβ (1 ng ml^−1^) for 48 h. To block TGFβ signalling, 5 µM SB431542 was added 1 h before TGFβ stimulation. Scale bars, 100 µm. (*b*) SCP4 accelerates TGFβ-induced migration. Confluent monolayers of MCF10A cells stably expressing SCP4 or GFP were scratched with a pipette tip to create a cell-free line. The wounded cell monolayers were incubated in OPTI-MEM medium with 1 ng ml^−1^ of TGFβ alone or in combination with inhibitor SB431542 for the indicated time periods. The wound healing was recorded by microphotography of the same region. Scale bars, 200 µm. (*c*) SCP4 enhances cell invasiveness. MDA-MD-231 cells stably expressing SCP4 or GFP were cultured in the transwell culture chambers with an 8 µm pore-size polycarbonate filter according to the manufacturer's instructions. Eight hours later, invaded cells that were attached to the lower surface of the filter were stained with DAPI. The slices were imaged. Scale bars, 200 µm. (*d*) SCP4 increases TGFβ-induced EMT marker expression. MCF10A cells stably expressing SCP4 or GFP were treated with 1 ng ml^−1^ TGFβ alone or in combination with SB431542 for 48 h. Levels of E-cadherin and Snail were analysed by western blotting. (*e*) SCP4 enhances TGFβ-induced E-cadherin mRNA inhibition. MCF10A cells stably expressing SCP4 or GFP were treated with 1 ng ml^−1^ TGFβ alone or in combination with SB431542 for 48 h. Total mRNA was extracted for RT–PCR analysis. Data are shown as mean ± s.e.m. (triplicate assays). *p < 0.05 SCP4 versus GFP. (*f*) SCP4 enhances TGFβ-induced fibronectin mRNA expression. Experiment was carried out as described for (*e*). Data are shown as mean ± s.e.m. (triplicate assays). *p < 0.05 SCP4 versus GFP. (*g*) SCP4 enhances TGFβ-induced vimentin mRNA expression. Experiment was carried out as described for (*e*). Data are shown as mean ± s.e.m. (triplicate assays). *p < 0.05 SCP4 versus GFP.

We previously identified SCP4 as a phosphatase for BMP-activated Smad1/5/8 in the BMP signalling pathway, but not Smad2/3 in the TGFβ signalling pathway [25]. Interestingly, we noticed that SCP4 could affect TGFβ-induced EMT in MCF10A cells. MCF10A, an immortalized human mammary epithelial cell line, has been demonstrated to undergo EMT upon exposure to TGFβ. Apparently, such EMT-promoting activity of SCP4 could be attributed to targeting other factors other than Smad proteins. In order to identify the SCP4 target in EMT, we first generated stable MCF10A cell lines either expressing SCP4 or GFP. Compared with control GFP-expressing cells, SCP4-expressing MCF10A cells appeared to grow less tightly in normal culture medium. Upon 48 h of 1 ng ml^−1^ TGFβ treatment, SCP4-expressing MCF10A cells began to lose their polarized epithelial cell morphology and became scattered and spindle-like, resembling mesenchymal cell morphology, which represents typical EMT morphological changes. In sharp contrast, control cells retained tight cell adhesion, indicating that the concentration of TGFβ used in this assay was able to distinguish the sensitivity of the two groups of cells for EMT induction. Moreover, these morphological changes could be reversed by SB431542, a specific inhibitor of TGFβ type I receptor (figure 1*a*).

To further confirm SCP4 can enhance TGFβ-induced EMT, we examined cell motility by wound healing and transwell assays. As shown in figure 1*b*, SCP4 accelerated the rate of cell migration in response to 1 ng ml^−1^ of TGFβ, which could be completely blocked with SB431542. By contrast, control GFP cells appeared to have no change under the same condition. This result is consistent with our observation that SCP4 induces morphological changes upon low-concentration TGFβ stimulation at a suboptimal concentration. We also tested whether SCP4 could enhance the motility of MDA-MB-231 cells, a triple negative human breast cancer cell line using transwell assay. As shown in figure 1*c*, the result showed that SCP4 greatly promoted cell migration even without exogenous TGFβ treatment.

We next examined the molecular changes of MCF10A in the process of EMT. E-cadherin is a well-known epithelial marker, while fibronectin and vimentin are typical mesenchymal markers. As expected, TGFβ treatment for 48 h decreased the level of E-cadherin and increased the level of Snail. Over-expressing SCP4 enhanced TGFβ-mediated reduction in E-cadherin reduction and increase in Snail (figure 1*d*). Consistent with western blot results, qRT–PCR analysis of EMT markers showed that SCP4 enabled a more profound decrease in E-cadherin mRNA in the presence of TGFβ (figure 1*e*), and dramatically increased the mRNA levels of fibronectin (figure 1*f*) and vimentin (figure 1*g*). Taken together, these results suggest that SCP4 may be an important player in regulating EMT.

### 3.2. Knockdown of SCP4 expression attenuates TGFβ-induced epithelial–mesenchymal transition

**Figure 2.**
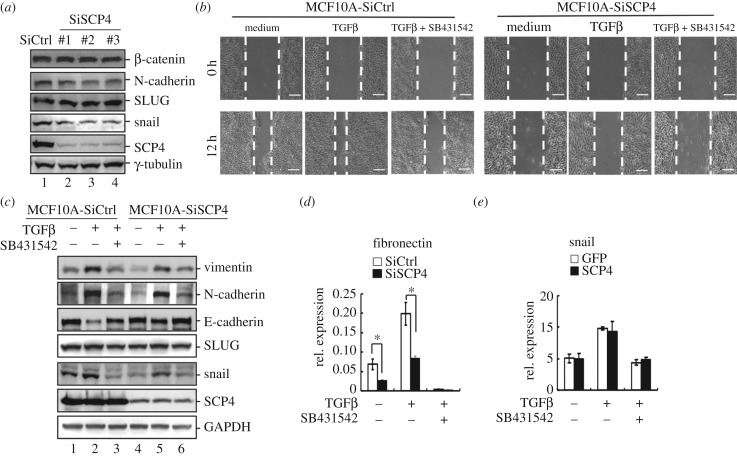
Depletion of SCP4 expression attenuates TGFβ-induced EMT. (*a*) Knockdown of SCP4 decreased the protein level of Snail. MCF10A cells were transfected with SCP4 siRNA (SiSCP4#1, #2 and #3) or control siRNA (SiCtrl). Whole cell lysates were harvested for western blotting analysis with β-catenin, N-cadherin, Slug, Snail, SCP4 and γ-tubulin antibodies. (*b*) Knockdown of SCP4 decreased TGFβ-induced cell migration. MCF10A cells were transfected with SCP4 siRNA (SiSCP4) or control siRNA (SiCtrl). Experiment was carried out as described for figure 1*b*. The wound healing was recorded by microphotography of the same region. Scale bars, 200 µm. (*c*) Knockdown of SCP4 attenuates TGFβ-induced EMT marker expression. MCF10A cells transfected with SCP4 siRNA (SiSCP4) or control siRNA (SiCtrl) were treated with 2 ng ml^−1^ TGFβ alone or in combination with SB431542 for 48 h. Whole cell lysates were harvested for western blotting analysis with E-cadherin, N-cadherin, vimentin, Slug, Snail, SCP4 and GAPDH antibodies. (*d*) Knockdown of SCP4 decreased TGFβ-induced fibronectin mRNA expression. MCF10A cells transfected with SCP4 siRNA (SiSCP4) or control siRNA (SiCtrl) were treated with 2 ng ml^−1^ TGFβ alone or in combination with SB431542 for 48 h. Total mRNA was extracted for RT–PCR analysis. Data are shown as mean ± s.e.m. (triplicate assays). *p < 0.05 siSCP4 versus siCtrl. (*e*) SCP4 has no effect on TGFβ-induced Snail mRNA expression. Experiment was carried out as described for (*d*).

To determine the physiological functions of SCP4 in EMT regulation, we examined the effect of SCP4 knockdown in TGFβ-induced cell migratory and EMT responses. Knockdown of SCP4 was achieved by three different SCP4 siRNAs: siSCP4#1, siRNA#2 and siRNA#3. When SCP4 was knocked down, the protein levels of Snail and N-cadherin were markedly decreased, while Slug, β-catenin and γ-tubulin were not influenced (figure 2*a*). Using these siRNAs to knock down SCP4 in MCF10A cells, we first carried out a wound-healing assay. As shown in figure 2*b*, TGFβ treatment (2 ng ml^−1^, 12 h) enabled a markedly enhanced migration of MCF10A cells. On the contrary, knockdown of SCP4 significantly attenuated TGFβ-induced migratory response in MCF10A cells. Moreover, by comparing expression of EMT marker expression, we found knockdown of SCP4 reduced the upregulation of mesenchymal markers (N-cadherin and vimentin) and downregulation of epithelial marker (E-cadherin). These results indicate that SCP4 regulates EMT under physiological conditions.

We also examined the effect of SCP4 knockdown on expression of EMT transcription regulators Snail and Slug. Notably, knockdown of SCP4 also decreased the expression of Snail, but not Slug (figure 2*c*), suggesting that SCP4 may promote EMT through regulating expression of Snail. We also measured the effect of SCP4 on the mRNA level of Snail. Interestingly, SCP4 knockdown inhibited the induction of fibronectin mRNA (figure 2*d*), so it was surprising that SCP4 knockdown had no effect on the mRNA level of Snail (figure 2*e*). These results support the notion that SCP4 regulates Snail through a post-translational mechanism.

### 3.3. SCP4 promotes Snail stabilization and prevents ubiquitination

**Figure 3.**
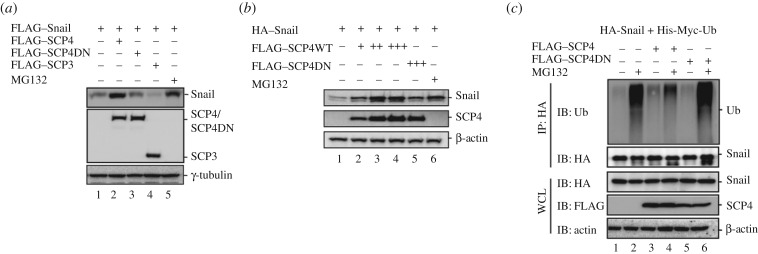
SCP4 prevents polyubiquitination and promotes stability of Snail. (*a*) SCP4 enhances Snail stabilization. HEK293T cells were co-transfected with FLAG–Snail and a FLAG tagged phosphatase. After 48 h later, cells were treated with or without MG132 for 6 h. The protein level of Snail was detected by western blotting analysis (*b*). SCP4 stabilizes Snail in a dosage-dependent manner. HEK293T cells were co-transfected with equal amount of FLAG–Snail and an increased dosage of SCP4 or SCP4DN. After 48 h, cells were treated with MG132 for 6 h. Whole cell lysates were harvested for western blotting analysis. (*c*) SCP4 inhibits Snail ubiquitination. HEK293T cells were co-transfected with HA–Snail, His-Myc-Ub and FLAG–SCP4/DN. After 48 h, cells were treated with or without MG132 for 6 h. Cells were lysed by RIPA lysis buffer and Snail was immunoprecipitated by anti-HA antibody.

Having determined the effect of SCP4 on regulating the Snail protein level, we reasoned that SCP4 impacts the stability of Snail. Snail is a highly unstable protein with a short half-life about 25 min [21]. To this end, we tested the effect of SCP4 on Snail stabilization in HEK293T cells. As expected, treatment with proteasome inhibitor MG132 significantly increased Snail protein level. Ectopic expression of SCP4 also dramatically stabilized Snail to a similar level to that done by MG132. By contrast, the phosphatase-dead mutant SCP4DN and SCP3 had no effect on Snail stabilization (figure 3*a*). These results indicate that SCP4 can stabilize Snail *in vivo*, and the stabilization is dependent on its catalytic activity. Next, we expressed Snail with wild-type SCP4 or phosphatase-dead mutant SCP4DN in HEK293T cells, and treated the cells with proteasome inhibitor MG132 to stabilize Snail. We found that Snail was stabilized to the same level after treating with MG132.

When Snail cDNA was co-transfected with increased amounts of SCP4 cDNA in HEK293T cells, we found that SCP4 caused Snail stabilization in a dosage-dependent manner (figure 3*b*). With high expression of SCP4, the Snail level reached the level induced by MG132, a proteasome inhibitor blocking protein degradation. In comparison, phosphatase-dead mutant SCP4DN exerted no effect on Snail stabilization (figure 3*b*). We also found that SCP4 could stabilize Snail in EMT-relevant MCF10A cells (data not shown).

As Snail degradation is mediated by the ubiquitin–proteasome pathway [21], we reasoned that stabilization of Snail could be attributed to decreased ubiquitination in the presence of SCP4. We thus examined the effect of SCP4 on Snail ubiquitination. HA–Snail was co-expressed with His-Myc-Ubiquitin to engage the ubiquitinaiton and then immunoprecipitated by HA antibody from cells treated with or without MG132. As expected, a high level of Snail ubiquitination was detected by anti-ubiquitin antibody in cells treated with MG132, which prevented proteasomal degradation of polyubiquitinated Snail. We found that wild-type SCP4, but not phosphatase-dead mutant SCP4DN, dramatically decreased Snail ubiquitination and stabilized Snail (figure 3*c*). Together, our results indicate that SCP4 stabilizes Snail through blocking its ubiquitination.

### 3.4. SCP4 dephosphorylates Snail

**Figure 4.**
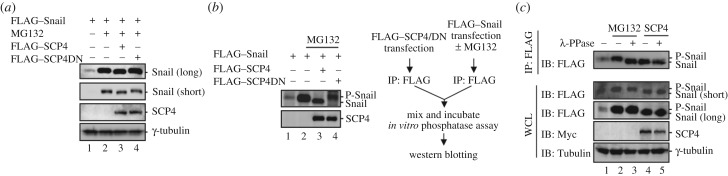
SCP4 dephosphorylates Snail. (*a*) SCP4 causes Snail a migration shift *in vivo*. HEK293T cells were co-transfected with FLAG–Snail and FLAG–SCP4 or FLAG–SCP4DN. After 48 h, cells were treated with or without MG132 for 6 h. The protein level of Snail was detected by western blotting analysis. (*b*) SCP4 causes Snail a migration shift *in vitro*. *In vitro* reaction assay was carried out as described in the schema at the right. HEK293T cells were transfected with FLAG–Snail (with or without MG132 treatment) or FLAG–SCP4/DN to express respective proteins. Cell lysates were harvested by RIPA lysis buffer (150 mM NaCl, 20 mM Tris–HCl (pH 7.5), 1% Triton X-100, 1% sodium deoxycholate, 0.1% sodium dodecyl sulfate). FLAG–Snail or FLAG–SCP4/DN proteins were purified by IP with anti-FLAG antibody, respectively. Purified FLAG–Snail and FLAG–SCP4 were incubated in *in vitro* phosphatase buffer at 30°C for 90 min. (*c*) SCP4 dephosphorylates Snail. HEK293T cells were co-transfected with FLAG–Snail and Myc-SCP4 or empty vector. After 48 h, cells were treated with or without MG132 for 6 h. Snail was immunoprecipitated from cell lysate and the immunocomplex was then incubated with or without λ-phosphatase for 30 min. Whole cell lysate and immunocomplex were subjected to western blotting analysis.

As already shown in figure 3*a*, we found that high expression of SCP4 stabilized Snail to the same level as MG132 treatment. Interestingly, we noticed that SCP4 caused Snail to migrate faster on an SDS–PAGE gel than vector control and SCP4DN, which occurred even in the presence of MG132 (figure 4*a*, lane 3). Because SCP4 is a phosphatase, this band shift might be due to Snail dephosphorylation. We then determined whether this band shift could be directly induced by SCP4 *in vitro*. SCP4 or SCP4DN was purified by anti-FLAG IP from HEK293T cells transfected with FLAG–SCP4 or FLAG–SCP4DN. Separately, Snail was purified from HEK293T cells transfected with FLAG–Snail and treated with or without MG132 (figure 4*b*, right). Immunopurified SCP4 (wild-type or phosphatase-dead mutant DN) and Snail were mixed and incubated in a phosphatase buffer, in which SCP4 retained its phosphatase catalytic activity. Figure 4*b* shows clearly that wild-type SCP4, but not the phosphatase-dead mutant SCP4DN, induced a faster migration of Snail compared to Snail only control *in vitro*. Collectively, these results suggest that the phosphatase catalytic activity of SCP4 is required for the Snail band shift and stabilization.

To further identify whether the migration shift is due to the dephosphorylation of Snail by SCP4, we immunoprecipitated Snail and treated with λ-phosphatase *in vitro* to wipe out all phosphorylation of Snail. Snail was stabilized by MG132 or co-expressed SCP4 as shown in figure 4*c*. Consistent with our previous finding, Snail stabilized by SCP4 migrated a little bit faster. After immunoprecipitating both forms of Snail and treating them with λ-phosphatase, we detected the faster migrating Snail as long as the lysate was treated with λ-phosphatase or co-expressed with SCP4. Obviously, the SCP4-dephosphorylated Snail could not be further dephosphorylated by λ-phosphatase. These results indicate that the band shift was mediated by the dephosphorylation of Snail from a phosphorylated state to a non-phosphorylated form. Taken together, SCP4 can dephosphorylate Snail *in vivo* and *in vitro*.

### 3.5. SCP4 stabilizes Snail depending on Snail dephosphorylation

**Figure 5.**
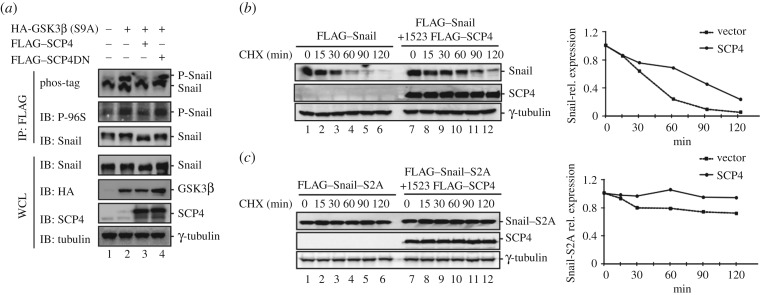
Dephosphorylation prolongs half-life of Snail. (*a*) SCP4 dephosphorylates Snail at GSK3β motif. HEK293T cells were co-transfected with FLAG–Snail, HA-GSK3β (S9A) and FLAG–SCP4/DN. After 48 h later, cells were treated with MG132 for 6 h. Snail was immunoprecipitated by anti-FLAG antibody and separated on SDS–PAGE or phos-tag gel and then subjected to western blotting analysis. (*b*) SCP4 prolongs half-life of Snail. HEK293T cells were co-transfected with FLAG–Snail and FLAG–SCP4 or empty vector. After 48 h later, cells were treated with CHX for 15, 30, 60, 90 or 120 min. Whole cell lysates were harvested for western blotting analysis. (*c*) SCP4 exhibits no effects on the half-life of Snail 2SA mutant. HEK293T cells were co-transfected with FLAG–Snail (2SA) and FLAG–SCP4 or empty vector. Snail (2SA) is a mutant with Ser-to-Ala substitutions at Ser-96 and Ser-100. Experiments were done as described in (*b*).

It has been previously reported that Ser-96 and Ser-100 of Snail in the N-terminal region are phosphorylated by GSK3β, which further induces the association of Snail with its E3 ligase β-TrCP, and thus leads to Snail degradation. To test whether SCP4 stabilizes Snail through these two sites, we immunoprecipitated Snail from cells treated with MG132, and detected the phosphorylation at Ser-96 and Ser-100 by using anti-phospho-β-catenin (Ser33/37/Thr41) antibody, which also recognizes these two phosphorylation sites [22]. To rule out the possibility that SCP4 dephosphorylates GSK3β to affect Snail phosphorylation, a constitutively active mutant of GSK3β (S9A) was used to stimulate Snail phosphorylation. As expected, the level of P-Snail increased in the presence of HA-GSK3β (S9A), and SCP4 apparently decreased the level of GSK3β(S9A)-induced P-Snail. By contrast, phosphatase-dead mutant SCP4DN exhibited no detectable effect on the phosphorylation level of Snail. We also tested the phosphorylation of Snail by using Phos-tag. As shown in figure 5*a*, Snail showed an upper phospho-band on Phos-tag gel only when cotransfected with HA-GSK3β (S9A). Wild-type SCP4, but not the phosphatase-dead mutant SCP4DN, completely diminished GSK3β (S9A)-induced phosphor-band. Collectively, these data clearly indicate that SCP4 dephosphorylates the GSK3β phospho-motif to keep Snail in a non-phosphorylated state, which leads to stabilization of Snail.

To further determine the effect of SCP4 on Snail stability, we examined the half-life of Snail. As shown in figure 5*b*, the protein level of Snail gradually decreased after treatment of a protein synthesis inhibitor cycloheximide (CHX), indicating a half-life of about 30 min. Co-expression of SCP4 could greatly suppress protein degradation and prolong its half-life to about 60 min. Because the phosphorylation of residues Ser-96 and Ser-100 is essential to Snail degradation, we also examined the effect of SCP4 on the mutant Snail-S2A (S96A, S100A). Compared with wild-type Snail, the mutant Snail-S2A showed much higher stability due to its resistance to further ubiquitination and SCP4 added no additional stabilizing effect (figure 5*c*). Taken together, SCP4 suppresses Snail degradation and prolongs its half-life in cells by phosphorylating the residues Ser-96 and Ser-100 on Snail proteins.

### 3.6. SCP4 directly interacts with Snail

**Figure 6.**
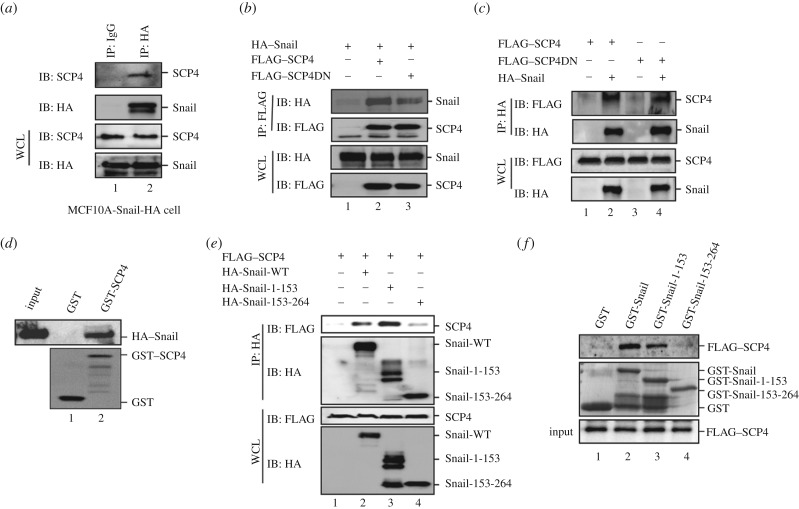
SCP4 physically interacts with Snail. (*a*) Endogenous SCP4 and Snail were co-immunoprecipitated in MCF10A cells. MCF10A cells stably expressing HA-Snail were harvested, and the lysates were immunoprecipitated with anti-HA antibody or control IgG. The co-IP complexes and the inputs were analysed by western blotting with the indicated antibodies. (*b*) SCP4 interacts with Snail *in vivo*. HEK293T cells were co-transfected with HA-Snail and FLAG–SCP4 or FLAG–SCP4DN. SCP4 was immunoprecipitated (IP) with anti-FLAG antibody and then subjected to SDS–PAGE and western blotting (IB) to detect SCP4 bounded Snail. WCL, whole cell lysate. (*c*) SCP4 interacts with Snail *in vivo*. HEK293T cells were co-transfected with HA-Snail and FLAG–SCP4 or FLAG–SCP4DN. Snail was immunoprecipitated (IP) with anti-HA antibody and then subjected to SDS–PAGE and western blotting (IB) to detect Snail-bounded SCP4. (*d*) Direct interaction between SCP4 and Snail *in vitro*. GST-SCP4 fusion protein was expressed in *E. coli* and purified by glutathione beads. *In vitro* translated HA-Snail was incubated with purified glutathione bead-bound GST protein or GST-SCP4. The retrieved complex was subjected to SDS–PAGE and western blotting analysis. (*e*) SCP4 interacts with the N-terminal region of Snail. HEK293T cells were co-transfected with FLAG–SCP4 and HA-Snail-WT or its deletion mutant HA-Snail-1-153, HA-Snail-153-264. Snail was immunoprecipitated (IP) with anti-HA antibody and then subjected to SDS–PAGE and western blotting (IB) to detect Snail bounded SCP4. (*f*) SCP4 directly interacts with the N-terminal region of Snail *in vitro*. Full length GST-Snail fusion protein or its deletion mutant GST-Snail-1-153 or GST-Snail-153-264 were expressed in *E. coli* and purified by glutathione beads. *In vitro* translated FLAG–SCP4 was incubated with purified glutathione bead-bound GST protein, GST-Snail, GST-Snail-1-153 or GST-Snail-153-264. The retrieved complex was subjected to SDS–PAGE and western blotting analysis.

As SCP4 dephosphorylated Snail and increased the stability of Snail, we speculated that SCP4 might directly bind to Snail. To test this, we carried out both *in vivo* and *in vitro* protein interaction experiments. First, we examined the interaction between endogenous SCP4 and Snail in MCF10A cells expressing HA–Snail. Endogenous SCP4 could be retrieved by anti-HA antibody IP, but not by control IgG IP (figure 6*a*). In addition, we performed a co-IP experiment with co-expressed HA–Snail and FLAG–SCP4 or its phosphatase-dead mutant SCP4DN in HEK293T cells. Results showed that anti-FLAG IP of either SCP4 or SCP4DN could retrieve HA–Snail (figure 6*b*). On a reverse co-IP assay, anti-HA (Snail) IP could similarly retrieve FLAG–SCP4 or FLAG–SCP4DN (figure 6*c*), indicating that SCP4 interacts with Snail independent of its phosphatase activity.

To rule out the possibility that SCP4 may indirectly regulate Snail, we generated recombinant GST–SCP4 fusion proteins and tested its association with Snail *in vitro* by GST-pulldown assay. As shown in figure 6*d*, GST–SCP4, but not GST alone, bound to Snail.

To further determine the region in Snail responsible for association with SCP4, two large deletions of Snail were generated based on its sequence features. The N-terminal region of Snail (amino acids 1–153) possesses the serine-rich residues, and the C-terminal region of Snail (amino acids 153–264) harbours the conservative zinc-finger motif. HA-tagged wide-type full-length Snail or two deletion mutants of Snail were co-expressed with FLAG–SCP4 in HEK293T cells. We found that immunoprecipitated SCP4 could retrieve the N-terminal region of Snail as well as full-length Snail. However, little association between the C-terminal region of Snail and SCP4 was noted, indicating that the N-terminal region of Snail is mainly responsible for the association with SCP4 (figure 6*e*). This result was further confirmed using *in vitro* binding analysis (figure 6*f*). These results demonstrate that SCP4 directly interacts with Snail by binding to its N-terminal region, which contains Ser-96 and Ser-100 residues for (de)phosphorylation.

## 4. Discussion

Snail is a critical transcriptional repressor of E-cadherin expression in this process, and its activity is tightly regulated at transcriptional and post-transcriptional levels. Normal epithelia do not express Snail; however, its transcription is induced in response to environmental EMT signals. TGFβ has been shown to increase Snail transcription during the wound healing process [28]. In addition to its transcriptional regulation, Snail is controlled by post-translational modifications. It is phosphorylated by GSK3β at Ser/Thr residues and degraded by β-TrCP-mediated ubiquitination. Wu *et al*. [24] tested 11 phosphatases and found that SCP1/2/3, but not FCP1 in the SCP/FCP phosphatase family, dephosphorylate Snail and enhance the activity of Snail. Here in this study, we added a new phosphatase targeting Snail for dephosphorylation and stabilization.

**Figure 7.**
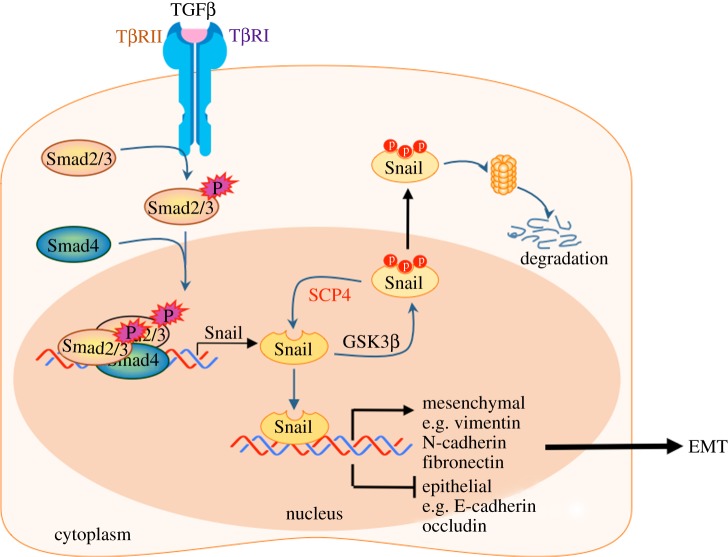
Working model of SCP4 as a novel Snail phosphatase. SCP4 dephosphorylates Snail, thereby suppressing the ubiquitin-dependent proteasome degradation of Snail and enhancing TGFβ-induced EMT.

During the study of protein phosphateome, we noted that ectopic expression of a novel phosphatase SCP4, a member of the SCP/FCP family, caused loose cell–cell contact and enhanced TGFβ-induced cell migratory response. In the current study, we report the identification and characterization of SCP4 for Snail stabilization. Subsequently, systematic experiments revealed SCP4 promotes TGFβ-induced EMT through dephosphorylating Snail at GSK3β phospho-motif. SCP4 is ubiquitously expressed in the nucleus and stabilizes nuclear Snail that further regulates its physiological functions (figure 7). Despite sharing a conserved phosphatase domain in FCP/SCP family, SCP4 shows a stronger effect than its relatives SCP1/2/3 in Snail dephosphorylation, which may be attributed to differential binding of SCPs to Snail. Interestingly, SCP4 interacts with N-terminal region of Snail, which differs from SCP1/2/3 that interacts with the C-terminal region of Snail.

Distinct properties of SCP4 underscore its importance in its specific role in regulating EMT. First, our study indicates that SCP4 specifically targets Snail in the TGFβ-induced EMT, but not Slug or β-catenin. Belonging to the same family, Slug is 48% identical to Snail in amino acid sequence and both of them share a common structure, with a highly conserved C-terminal domain and a divergent N-terminal region [29,30]. In N-terminal region, Slug contains the so-called SLUG domain, required for efficient Slug-mediated repression, while Snail presents a regulatory domain containing a nuclear export signal and a destruction box domain. These differences make Slug not be the target of SCP4 like Snail. β-Catenin shares the same destruction box domain with Snail. Like Snail, β-catenin can be phosphorylated by GSK3β, and β-TrCP promotes its ubiquitination and proteasomal degradation [31,32]. However, phosphorylated β-catenin was degraded in the cytoplasm, and only when the activity of GSK3β is inhibited will β-catenin be stabilized and transported into the nucleus. Obviously, nuclear phosphatase SCP4 cannot dephosphorylate and stabilize β-catenin in the cytoplasm.

It has recently been reported that SCP4 negatively shuts off BMP-induced signalling in a phosphatase-dependent manner, whereas it has no effects on TGFβ-induced transcriptional regulation. SCP4 does so by specifically targeting Smad1/5/8 in the BMP pathway, but not Smad2/3 in the TGFβ pathway. Here, we added a new EMT-promoting function to SCP4. Although TGFβ drives EMT, it is apparent that SCP4 promotes not through regulating Smad2/3, but by dephosphorylating Snail. SCP4 consequently prevents ubiquitination of Snail and stabilizes Snail protein, thereby promoting TGFβ signalling in EMT.

## Data accessibility

All relevant data are presented in the figures in the manuscript.
